# A First-Tier Framework for Assessing Toxicological Risk from Vaporized Cannabis Concentrates

**DOI:** 10.3390/toxics10120771

**Published:** 2022-12-09

**Authors:** Shawna Vreeke, David M. Faulkner, Robert M. Strongin, Echoleah Rufer

**Affiliations:** 1 Product, True Terpenes, Hillsboro, OR 97124, USA; 2 Health Sciences, PAX, San Francisco, CA 94110, USA; 3 Department of Chemistry, Portland State University, Portland, OR 97207, USA

**Keywords:** cannabis, vaporization, vape, exposure, toxicology, risk assessment framework, cannabis concentrate, cannabis additives

## Abstract

Vaporization is an increasingly prevalent means to consume cannabis, but there is little guidance for manufacturers or regulators to evaluate additive safety. This paper presents a first-tier framework for regulators and cannabis manufacturers without significant toxicological expertise to conduct risk assessments and prioritize additives in cannabis concentrates for acceptance, elimination, or further evaluation. Cannabinoids and contaminants (e.g., solvents, pesticides, etc.) are excluded from this framework because of the complexity involved in their assessment; theirs would not be a first-tier toxicological assessment. Further, several U.S. state regulators have provided guidance for major cannabinoids and contaminants. Toxicological risk assessment of cannabis concentrate additives, like other types of risk assessment, includes hazard assessment, dose–response, exposure assessment, and risk characterization steps. Scarce consumption data has made exposure assessment of cannabis concentrates difficult and variable. Previously unpublished consumption data collected from over 54,000 smart vaporization devices show that 50th and 95th percentile users consume 5 and 57 mg per day on average, respectively. Based on these and published data, we propose assuming 100 mg per day cannabis concentrate consumption for first-tier risk assessment purposes. Herein, we provide regulators, cannabis manufacturers, and consumers a preliminary methodology to evaluate the health risks of cannabis concentrate additives.

## 1. Introduction

As of October 2022, 38 states and the District of Columbia permit cannabis in some capacity, for adult use or medical purposes, but cannabis remains federally illegal. Divergence in state and federal cannabis regulations creates challenges for state-legal cannabis manufacturers. Cannabis concentrate (also referred to as cannabis oil or cannabis extract) vaporization products (i.e., “vape pens”) are one of the fastest growing cannabis segments and often a starting point for new consumers. As the cannabis industry develops, regulators and cannabis manufacturers alike need actionable methods for toxicological risk assessment of inhalable cannabis concentrate additives. Additives in cannabis concentrates can pose a range of toxicological risks, from E-cigarette, or Vaping Product, Use Associated Lung Injury (EVALI) in illicit vape products to increased risk of carcinogenicity from terpenoid compounds (e.g., pulegone) to minor respiratory irritation or negligible effect. This manuscript aims to reduce consumer risk by providing a first-tier framework accessible to non-toxicologists to help manufacturers and regulators conduct preliminary evaluations with data available today to prioritize cannabis extract additives for acceptance, elimination, or further evaluation.

The scope of this manuscript includes ingredients intentionally added (natural and synthetic, excluding cannabinoids) to THC-containing cannabis extracts (also referred to as concentrates) intended to be vaporized. Both natural and synthetic compounds are included because the source of a compound is not inherently tied to risk. Natural pesticides used in organic farming are just as toxic as synthetic pesticides, and many known toxicants are entirely natural [1,2]. Additionally, this manuscript does not differentiate between naturally extracted (from cannabis or other botanicals) and synthetically made versions of the same compound, because natural and synthetic compounds produce identical biological effects so long as their chemical structure and spatial orientation are identical. This concept is also borne out in U.S. federal regulation as the U.S. FDA considers “Highly purified substances, either derived from a naturally occurring source or chemically modified” relevant to its guidance for botanical drug development [3]. In other words, the purity of a compound rather than the source drives the effect.

Cannabinoids are excluded from this framework for two reasons. The first is that, due to its complexity, cannabinoid assessment is not a first-tier assessment. The second is that several US state regulators have provided guidance for the major cannabinoids. Exclusion from this manuscript does not mean risk assessment of cannabinoids is unnecessary or that they are risk-free, only that they are beyond our present focus. It is important to identify contaminants (in the cannabis concentrate and the aerosol that is produced when the cannabis concentrate is vaporized) to determine the toxicological risk. However, this was excluded from this first-tier framework due to the complexity of the assessment, likely lower exposure than intentionally added ingredients, and the lack of availability for commercial test labs to test cannabis concentrate aerosol. In addition, regulatory frameworks are already available in some jurisdictions to address contaminants in cannabis concentrate products [4].

In 2019, an outbreak of a lung injury called EVALI was first identified and eventually lead to over 2800 hospitalizations and 68 confirmed deaths due to use of THC-containing vaping products acquired from illicit, informal sources [5]. Research has since shown that the additive, vitamin E acetate, is strongly linked with EVALI highlighting the need to urgently develop reasonably accessible ways to protect public health [5,6]. This manuscript is intended to begin the conversation about toxicological risk assessment methods for inhalable cannabis concentrate additives that can meet the needs of regulators and manufacturers seeking to improve cannabis product safety.

Traditional toxicological risk assessment follows a template: (1) Identify the potential hazard of a substance (the innate capacity of a substance to cause harm); (2) Determine the dose at which harmful effects are expected to occur (the point of departure, or POD); (3) Identify the extent of exposure to a population of interest; and finally, (4) Use knowledge from the previous steps to characterize the risk to the population of interest. Risk assessors generally take a tiered approach so that the assessment only goes as far as is needed. If the substance is low risk, the assessment may conclude early to save time and resources that may be required for more advanced toxicological risk assessments. This practice is used by the U.S. Environmental Protection Agency (EPA) to evaluate environmental chemicals, the European Food Safety Authority (EFSA) to evaluate food ingredients, and the World Health Organization (WHO) to optimize the use of limited resources needed to prioritize chemicals for evaluation [7,8,9]. In fact, only 21.6% of the chemicals in the American food supply have sufficient data to estimate a safe level of exposure [10].

In the last few decades, regulatory bodies have shifted their approaches to evaluating chemicals by using and accepting new approach methodologies (NAMs) and exploring ways to prioritize substances more effectively for more in-depth toxicological evaluation [11,12]. NAMs include computational models, the threshold of toxicological concern (TTC), in vitro methods, read across, and others. A consequence of the current cannabis regulatory environment in the U.S. is that many cannabis manufacturers will have to rely on NAMs.

As with other product categories, few cannabis concentrate additives have been thoroughly tested for safety by all exposure routes (e.g., inhalation, oral, and dermal). In the case of cannabis products, it is critical for regulators and industry operators to strike a balance between prudently testing and assessing cannabis products and pushing state-legal cannabis product manufacturers to the illicit market. In the U.S., the illegal market is estimated to be two to three times the size of the legal market and is neither regulated nor compelled to comply with regulations [13].

To date, more research has been done on nicotine product vaping than cannabis vaping, but conclusions from e-cigarette research should only be extrapolated to cannabis with caution and appropriate adjustments. There are significant differences between electronic nicotine delivery systems (ENDS) and cannabis vaping products that may affect the toxicological risk: (1) Higher temperatures: cannabinoids have higher boiling points and are more viscous than nicotine, requiring higher temperatures for vaporization. This raises concerns about increased thermal degradation in cannabis vaporizers, although vaporization temperatures are still significantly lower than those encountered in combustion smoking. (2) Cannabis formulations are fundamentally different from ENDS due to inherent differences in potency and absorption between nicotine and cannabinoids. ENDS e-liquids are mostly solvents (propylene glycol and vegetable glycerin up to 90% by weight) with 1.5–5% nicotine and 0.1–10% flavor mixture [14]. Solvents used in ENDS may produce harmful degradation products such as formaldehyde and other carbonyls when exposed to elevated temperatures [15,16,17]. In contrast, current cannabis vaporization concentrates in the U.S. and Canadian regulated markets typically contain 60–90% cannabinoids (such as THC and cannabidiol (CBD)) and 5–15% terpenoids or flavors, with the remainder comprised of other cannabis compounds pulled through during the extraction process [18,19,20]. Often, these added terpenoids are collected during an additional step in the cannabinoid extraction that otherwise would have been lost due to the higher temperature required for cannabinoid extraction [21]. Because THC concentration drives value for the regulated-market consumer and must be reported on the label, large solvent concentrations that reduce THC concentrations are not used by legal, reputable cannabis extract manufacturers [22]. CBD products containing less than 0.3% THC are generally not part of the same regulated cannabis market as THC. Due to the chemical properties of CBD, it crystallizes at concentrations >~40% without other cannabinoids; some may contain solvents to allow the product to be vaporized. (3) As will be shared here, data show that daily usage is significantly lower than e-cigarette usage—up to 10-fold lower. Because dose not concentration drives risk, this implies toxicological risk from additives may be lower for cannabis concentrate products than ENDS.

The lack of scientific data concerning the health risks of modern inhalable cannabis products needs to be addressed to inform regulators, consumers, manufacturers, and clinicians; however, a balanced approach is necessary to slow the illicit market, which cannot be monitored and is already a significant problem in the U.S. This framework may be used as a first approach to identify high-risk ingredients and prioritize substances for further evaluation.

## 2. Materials and Methods

PAX^®^ Era devices use advanced telemetry and a connected application that allows consumers to opt-in to share data with PAX. For those who have opted in to sharing usage data, anonymized device usage data collected from November 2020 to May 2022 has been analyzed to determine H&P. Daily usage (in mg) was estimated from controlled laboratory studies by weighing the pod before and after a puff with various puffing parameters to simulate the breadth of user settings. A proprietary machine learning algorithm was trained on the laboratory data to predict the mass of aerosol product per puff based on device-logged telemetry data. A comprehensive range of machine learning algorithms were trained on the laboratory data. The model with the lowest cross-validated root mean squared error (RMSE) was selected for prediction. The model was then applied to 90,000 user-shared puffs to estimate consumption patterns in the field. The error rate for this method is approximately 20% due to different user H&P than what was used to validate the model. This error compounds at higher consumption levels. Importantly, these data are based on aerosol produced by a single device type and assume that device is used by one person and that each consumer uses only one device. Cannabis consumption is often consumed socially so it is likely that some of these devices are used by more than one individual and therefore somewhat overestimating daily exposure.

## 3. Results

### 3.1. Gathering the Data (Hazard Identification)

**Figure 1:**
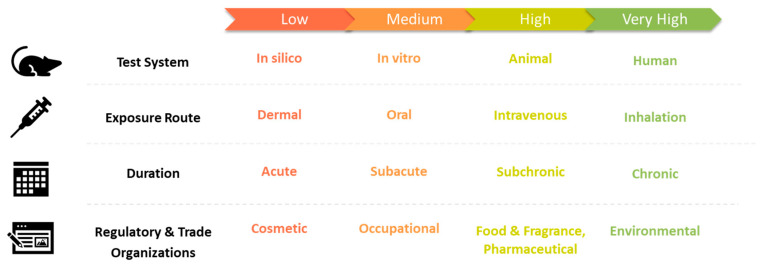
Relevance weights of studies for risk assessment of inhalable cannabis concentrate additives. Higher weighted data shall contribute greater weight to the risk assessment.

Toxicological data and safety assessments from all sources must be considered, including clinical, epidemiological, in vivo, in vitro, in silico, and thermal degradation studies. Publicly available guideline studies (e.g., European Chemical Agency (ECHA) dossiers (https://echa.europa.eu/information-on-chemicals/registered-substances accessed on 27 October 2022), Research Institute on Fragrance Materials (RIFM) assessments (http://fragrancematerialsafetyresource.elsevier.com accessed on 27 October 2022)) and peer-reviewed publications should be collected from databases such as Toxplanet.com (Arlington, VA, USA), U.S. National Institutes of Health (NIH)’s PubMed (https://pubmed.ncbi.nlm.nih.gov, accessed on 27 October 2022), ScienceDirect (https://www.sciencedirect.com, accessed on 27 October 2022), Google Scholar (https://scholar.google.com, accessed on 27 October 2022), and others. All toxicological endpoints must be considered, including systemic toxicity (e.g., liver, kidney, neurotoxicity, immunotoxicity, etc.), local effects on the respiratory tract, developmental and reproductive toxicity, and carcinogenicity (including genotoxicity and mutagenicity). The reliability of each study should be evaluated based on guideline compliance (e.g., good laboratory practices (GLP), good clinical practices (GCP), OECD guidelines), quality (e.g., Klimisch score), and relevance of each study. The U.S. EPA provides guidelines on evaluating open literature for risk assessment purposes [23]. Each study should be weighted based on quality and applicability, as shown in Figure 1, with the most relevant given the highest weight. Thermal degradation studies (e.g., pyrolysis, reactive oxygen species formation) are valuable because cannabis extracts are vaporized at temperatures as high as or higher than 600 °C in some vaporization systems [24]. Some substances have been studied in ENDS, and those data may also be relevant.

In some cases, regulatory restrictions on concentration or exposure by other industries may also be relevant. Since regulatory guidance is sparse for inhalable cannabis concentrate additives, regulations from other sectors can be used as a starting point with adjustments applied as needed. Some examples include U.S. FDA for food and pharmaceuticals, International Fragrance Association (IFRA) standards (https://ifrafragrance.org/safe-use/library accessed on 27 October 2022), U.S. EPA and state environmental regulators (e.g., Texas Commission on Environmental Quality (TCEQ, https://www17.tceq.texas.gov/tamis/index.cfm?fuseaction=home.welcome accessed on 27 October 2022), California Office of Environmental Health Hazard Assessment (OEHHA, https://oehha.ca.gov/air/general-info/oehha-acute-8-hour-and-chronic-reference-exposure-level-rel-summary accessed on 27 October 2022)) for permissible exposure limits and safe harbor levels, and global guidance for workplace exposure (e.g., ACGIH, OSHA, NIOSH). Concentration limits should be converted to exposure and not used directly. While occupational limits are often set for inhalation exposure, they may not be sufficiently protective for cannabis extract consumers. Occupational guidelines may be based on minimal data and do not guarantee that exposures occurred at the guidance limits so they may not provide much additional information. Further, the levels set in occupational guidance apply to generally healthy individuals [25]. Some cannabis extract consumers (especially medical cannabis patients) may be more susceptible to harmful effects due to a medical ailment and the direct application of occupational limits would not be recommended. Importantly, many occupational exposure limits are calculated based on constant exposure over an 8- or 10 h workday, five days per week; environmental air quality limits typically assume exposure for 24 h per day, seven days a week. When available, the short-term (15 min) exposure limit (STEL) should be used to derive the safety limit since the high concentration exposure over a short time is more similar to cannabis concentrate vaporization exposure. Either exposure limit may be significantly greater than cannabis concentrate consumers’ exposure. For example, the Nordic countries have derived a time-weighted average threshold limit value (TWA-TLV) of 25 ppm (135 mg/m^3^) for para-cymene [26]. Considering 6.7 m^3^ inhaled volume of air over 8 h [27,28,29], the OSHA limit would be equivalent to an exposure of 904.5 mg of para-cymene on a workday. As we’ll show in later sections, that is significantly greater than the total mass of extract vaporized by a 95th percentile consumer per day and suggests the toxicological risk is lower. The extended risk analysis on para-cymene is shown as Case Study S2 in the Supplementary Materials.

The Flavor and Extract Manufacturers Association of the U.S. (FEMA) has rated many substances used in cannabis extracts as “generally regarded as safe (GRAS) for use in foods” under certain defined conditions; however, these assessments are not directly applicable to vaping products. This is because inhalation bypasses the gastrointestinal tract and liver metabolizing and detoxifying enzymes, affording direct dosing without breakdown [30]. Although the lungs possess chemical defenses, the respiratory tract is much more sensitive than the gastrointestinal tract and it is unclear whether and to what extent the lungs can protect from vaping-related exposures. GRAS rating is not directly applicable to inhalation products, but safety data used for the GRAS determination can be incorporated into a toxicological risk assessment as a POD for systemic endpoints and with appropriate uncertainty factors applied. In the future, more advanced models (e.g., physiologically based kinetic (PBK) models) will allow more specific oral-to-inhalation extrapolation, but these are rarely available at this time for cannabis concentrate additives.

If data on the substance of interest is insufficient to calculate a safe level, applicable analogs may inform on toxicological risk [31,32,33]. This methodology, called read across, uses data from data-rich substances to assess the safety of a data-poor substance with sufficient similarity. Similarities are evaluated based on functional chemical moieties, physical-chemical properties, reactivity, toxicokinetics, mode of action, and toxicodynamics and should only be performed by an expert. This would not be part of a first-tier assessment. If exposure is very low, the threshold of toxicological concern (TTC) may be acceptable, as discussed in a later section.

Lastly, if the compound occurs in consumable portions of the cannabis plant, that concentration can be used as part of the weight of evidence to determine toxicological risk. Cannabis has been utilized medically, recreationally, and spiritually for millennia with fewer adverse effects identified than tobacco, many pharmaceuticals, and alcohol [34,35,36]. Even though the plant has changed through advanced breeding techniques, serious adverse events remain rare [37,38,39]. A recent systematic review of adverse events reported in controlled trials with cannabinoid medicines found no difference from control for serious adverse events; non-serious (most commonly dizziness) adverse events were 1.86 times higher than control [39]. Additional studies are warranted, but a recent analysis failed to find a difference in adverse event reports between smoked cannabis flower and cannabis concentrates, although sample size may have been insufficient [40]. Compare this to aspirin with a risk ratio of 1.6 vs. control, 2.2 for ibuprofen used with caffeine, and 5.3% of global deaths attributed to alcohol and 20% of U.S. deaths attributed to cigarette smoking [35,41,42]. Additionally, consumers tend to self-titrate inhaled THC doses [43,44]; therefore, the compounds at natural levels in the inflorescence relative to THC concentrations can be used in the risk assessment. The concentration of compounds in the cannabis plant can be identified from scientific literature and accredited cannabis testing labs [45]. While not risk-free (and assuming the product is compliant with cannabis contaminant limits and being used by adults as intended), vaporizing cannabis likely poses less health risk than cannabis combustion (i.e., smoking “joints”) due to the reduction of toxic compounds caused by thermal degradation at high temperatures [46,47,48,49]. This is important for many cannabis consumers who see vaporization as a harm reduction tool so there may be cases where some toxicological risk (if it is less than smoking cannabis) may be acceptable. Of course, more research is needed on the long-term health effects of cannabis concentrates themselves.

### 3.2. Dose Response Assessment

Before conducting a dose–response assessment of cannabis concentrate additives, there are several important types of toxicants that should be completely excluded, including non-cannabis respiratory sensitizers, carcinogens and genotoxicants, and certain chemical classes. Once these have been evaluated, the dose response assessment can commence to define the safety limit based on the dose at which harmful effects occur.

#### 3.2.1. Respiratory Sensitizers

Respiratory sensitizers can elicit severe reactions such as asthma, hay fever-like symptoms, and anaphylaxis. Unfortunately, there are currently no tests or risk assessment methodologies to determine respiratory sensitization risk, so conservative assumptions will be necessary to reduce consumer risk. Respiratory sensitization data suitable for a first-tier assessment are often lacking, but hazard classifications on Safety Data Sheets (SDSs) or publicly available on the ECHA website (https://echa.europa.eu/information-on-chemicals, accessed on 27 October 2022) and the Association of Occupational and Environmental Clinics (AOEC) databases (http://www.aoecdata.org/expcodelookup.aspx, accessed on 27 October 2022) can be used to classify an ingredient as a respiratory sensitizer for a first-tier assessment.

Respiratory sensitizers that do not naturally occur in cannabis should be entirely avoided in inhalable products because of the effect severity and extremely low safety limits. As an example, conservatively considering 0.3 m^3^ 15 min inhalation volume and a threshold of 0.002 mg/m^3^ for respiratory sensitization (based on the ACGIH 15 min STEL for trimellitic anhydride, a potent respiratory sensitizer), just 0.6 µg/day could pose a greater risk for an allergic response than for an individual working with this chemical. Possibly sensitizing proteins from essential oils, natural extracts, and direct use or contamination with the “Big 8” food allergens (eggs, Crustacean shellfish, peanuts, tree nuts, milk, fish, wheat, and soybeans) should also be avoided. Allergies to the “Big 8” are common and may elicit severe reactions when inhaled or consumed orally.

For cannabis, respiratory sensitization has been reported, albeit rarely [50]. Based on this historical use and likely exposure levels, respiratory sensitizers native to the cannabis plant are unlikely to pose an acute risk of anaphylaxis although other respiratory sensitization symptoms are possible. Respiratory sensitizer concentration of compounds native to the cannabis plant should be minimized and always kept below that from the cannabis plant relative to THC concentration [51].

#### 3.2.2. Genotoxic, Mutagenic, and Carcinogenic Substances

Genotoxic, carcinogenic, and mutagenic substances should be avoided, or appropriate TTC limits for genotoxic and carcinogenic substances should be applied (see below). Ideally, genotoxic and carcinogenic substances should be avoided entirely. This determination can be made based on data as shown in Figure 1 or regulatory classifications provided in the SDS (e.g., International Agency for Research on Cancer (IARC), Carcinogenic, Mutagenic, Reprotoxic (CMR) classifications, OEHHA Proposition 65 list, etc.).

#### 3.2.3. Other Substances to Avoid

Phenolic acetate compounds, herbal and dietary supplements, vitamins, minerals, and harmful and potentially harmful constituents (HPHCs) as identified by the U.S. FDA, Health Canada, and others should be avoided due to their pharmacological activity and toxicity potential.

It is widely reported that vitamin E acetate in illicit market THC vaporization products was responsible for the EVALI outbreak in 2019 and 2020 [5,6,52]. Research has since demonstrated several likely mechanisms for EVALI, such as alteration in lung surfactant and production of the potent respiratory toxicant ketene released from the phenolic acetate moiety of vitamin E acetate [53,54,55,56]. As such, vitamin E acetate should be entirely avoided. Other substances containing phenolic acetate moieties should be avoided unless sufficient data are available to show their lack of toxicity when vaporized, and a higher tier risk assessment is completed to determine toxicological risk in a cannabis concentrate vaping context.

Herbal supplements, drugs, and nutrients should not be added to a vaporization product without sufficient safety testing or a history of inhalation in a similar vaporization context. Orally consumed vitamins and herbal supplements such as melatonin, vitamin B12, St. John’s Wort, and caffeine could be toxic by inhalation. Their pharmacologic activity, along with possibly higher exposures caused by bypassing first-pass liver metabolism combined with exposure via other routes (i.e., taking large doses orally), could pose a significant risk to consumers. In Canada, these ingredients are not allowed in cannabis concentrate vaporization products.

### 3.3. Calculating a Safety Limit

Assuming that the ingredient of interest is not one of the excluded substances, the most sensitive effect is identified from data gathered during the hazard and dose–response assessment phases. The no observable adverse effect level or concentration (NOAEL or NOAEC) or other point of departure (POD) identified from that study is used to define the exposure which would pose a low toxicological risk to consumers. Importantly, LD50 and LC50 are not appropriate PODs in toxicological risk assessment [57,58]. Typically, toxicological data for a particular substance is not generated for every possible scenario; instead, safety factors (or uncertainty factors (UF)) are added to extrapolate between scenarios. It should be no different for cannabis products. UFs account for uncertainty when deriving occupational exposure limits, environmental limits, and others [8,25,59]. Six key uncertainties must be evaluated in a first-tier assessment (Table 1). UF categories include: extrapolating from animal studies to humans (interspecies), differential sensitivity among the human population (intraspecies), extrapolating from shorter duration studies to longer exposure in humans, extrapolation from non-inhalation routes to account for toxicokinetic (i.e., how the substance is absorbed, distributed, metabolized, and excreted from the body) and toxicodynamic differences (i.e., the biological effect(s) of the substance), uncertainty in estimating a NOAEL from a dose where adverse effects were observed (i.e., LOAEL), and uncertainty when developmental toxicity data are not available. A NOAEL should only be used if it is lower than all LOAELs; otherwise, the lowest LOAEL must be used (along with appropriate UFs). In this case, further assessment by an expert to determine the applicability of the effect observed may be necessary. Regulatory limits may already consider some areas of uncertainty. Those that have not been appropriately addressed must be added.

#### 3.3.1. Local Respiratory Toxicity

Where there is evidence that a chemical causes local respiratory toxicity (e.g., irritation of the respiratory tract), extrapolation from the oral route should be performed only for systemic effects. Local respiratory toxicity should be evaluated separately, and the more sensitive endpoint (i.e., lower limit) should be used. Some substances may not cause systemic toxicity (i.e., organs outside of the respiratory tract), but can cause toxicity to the respiratory tract when inhaled. In vitro testing (i.e., testing respiratory tract cells in a dish) of a finished product or ingredient can help understand local respiratory tract toxicity risk. While it does not guarantee that local toxicity will not occur, it does increase confidence in the assessment. Published studies (human, animal, and in vitro) conducted on individual ingredients and ENDS formulations are sometimes available to understand local respiratory tract toxicity. Substance concentrations should be kept below cytotoxic levels (i.e., levels that cause cell death) observed in vitro. For in vitro studies, it is especially important to consider the study design. For example, volatile compounds will evaporate from cell cultures so only limited concentrations can be reached in the cell culture media. Issues like solubility in cell culture media and cell type used are also important considerations. If the study of interest is not a guideline study in relevant cell types, consultation with an experienced toxicologist is recommended to determine the applicability of the in vitro study being considered. The final formulation may affect local respiratory tract toxicity, so additional testing may be necessary to make this determination. Unfortunately, in vitro testing of vaporized cannabis products containing THC is not feasible for most U.S. cannabis manufacturers, since few labs (if any) exist in the U.S. that are able and willing to do this type of testing and commercial cannabis products cannot be legally shipped across state lines.

#### 3.3.2. Developmental Toxicity

Considering the potential for developmental toxicity, cannabis products should only be used by pregnant women, children, and adolescents in exceptional situations under a physician’s guidance where the benefits and risks can be appropriately weighed [64,65,66]. For THC-containing products in the state-legal markets, there are several risk-mitigation strategies in place including warning labels against use during pregnancy, strict age-gating, and physician supervision [67,68]. The conservatism included in this framework may also provide additional protection for these products. Products that contain less than 0.3% THC (e.g., CBD-dominant) are legally permitted for use by adults 18 years and older and may not have the same risk mitigation strategies that are in place for THC-containing products; therefore, developmental toxicity risk should be more closely evaluated. Additional UFs (Table 1, category 6) are required if data are not available to determine a substance’s developmental toxicity potential [63].

#### 3.3.3. Calculating a Safety Limit with Available Data

The total uncertainty factor should not exceed 10,000 [25,69]. If it does, there is likely insufficient information to conduct a risk assessment, and other methods (e.g., TTC, more advanced toxicological risk assessment methods) will be needed. The first-tier safety limit is defined by Equation (1). An easy-to-use Safety Limit Assessment Calculator (SLAC) is included in the Supplementary Materials for various POD types.

(1) $$\mathrm{Safety}\;\mathrm{limit}=\frac{\mathrm{POD}}{\mathrm{UF}1\;\times \;\mathrm{UF}2\;\times \;\mathrm{UF}3\;\times \;\mathrm{UF}4\;\times \;\mathrm{UF}5\;\times \;\mathrm{UF}6}$$

where: 

Safety limit: The exposure at which risk is expected to be low. Units will be dependent upon the units used for the POD. For example, if the NOAEL is mg/kg body weight per day, the resulting safety limit will be in the same units.

POD (Point of Departure): The NOAEL or NOAEC identified during the hazard assessment process. This should be a concentration (e.g., mg chemical per kg body weight per day or mg chemical per m^3^ inhaled over a 24 h period in the animal tested) during the study. If exposure to the test species is not continuous (i.e., 24 h per day, seven days a week), then the POD needs to be adjusted (e.g., multiply the NOAEL by 5/7 if exposure occurs only five days per week, inhalation exposures conducted for 6 of 24 h for five days per week would need to have the NOAEC multiplied by 6/24 and 5/7). The POD should also be adjusted by purity. See the Case Study S1 in the Supplementary Materials for an example.

UF: As defined in Table 1. These are unitless.

#### 3.3.4. Threshold of Toxicological Concern

While inhalation toxicity data would be preferred, the reality is that few substances have inhalation toxicology data. When insufficient toxicological data are available to estimate a safety limit, threshold of toxicological concern (TTC) limits may be used. TTC values are generalized exposure limits used for chemicals that have little or no available toxicological data [70]. Importantly, TTC is accepted by the U.S. FDA and EMA for pharmaceuticals [71,72,73]. TTC is based on a large group of chemicals with toxicological data that have been categorized based on factors driving toxicity. The most toxic substances (5th percentile) in each category form the basis for the TTC limit of that category. Chemicals with unknown toxicity are then fit into the appropriate category and are assumed to be the most toxic in that category. Table 2 shows the questions that need to be considered to determine the appropriate TTC limit for compounds in vaporized cannabis extracts. Chemicals that do not fit appropriately into a category are excluded from TTC (Table 2, Question 1). To determine which TTC value applies, it first must be determined whether a compound is an organophosphate or has genotoxic potential (Table 2, Question 2 and 3). Non-genotoxic compounds are classified based on decision trees related to chemical and physical properties that affect the compound’s toxicity. Classifications can be made by entering the CAS registry number or other identifying information into freely available applications like ToxTree (https://apps.ideaconsult.net/data/ui/toxtree, accessed on 27 October 2022) and OECD QSAR Toolbox (http://www.qsartoolbox.org, accessed on 27 October 2022). It is important to note that while these models are useful, they do have important limitations. In general, multiple models should be run and the more conservative values chosen for a first-tier assessment. TTC limits are intentionally conservative and if exposure exceeds TTC, there are three options: (1) remove the additive or reduce the concentration, (2) conduct a more refined risk assessment, and (3) produce the data needed to conduct a full toxicological risk assessment.

Carthew et al. [74] calculated TTC values for inhaled chemicals using inhalation data. More recently, Nelms and Patlewicz [75] derived a new set of respiratory TTC limits that were more conservative than Carthew et al. (2009) derived from a larger set of chemicals that included pesticides, industrial chemicals, and other substances unlikely to be intentionally added to cannabis extracts. Given that the compounds typically used in cannabis extracts are allowable for use in food (and therefore are not likely to be potent toxicants), Carthew TTC values (adjusted by Nelms & Patlewicz) are more appropriate for many cannabis concentrate assessments than the environmental contaminants and industrial chemicals used by Nelms & Patlewicz. If the additive is not a GRAS or used in food then the Nelms & Patlewicz limits should be applied as indicated in Table 2. See Case Study S3 in the Supplementary Materials for an example.

### 3.4. Exposure Assessment

Understanding exposure is critical for understanding risk. Typically, 75th to 97.5th percentiles are used for estimating exposure for toxicological risk assessment purposes [27,76,77]. Importantly, not all parameters (i.e., body weight + daily consumption + absorption + lifetime use, etc.) should be a high percentile or else compounding conservatism can make the assessment unrealistic [78,79]. Considering this conservatism, a 95th percentile exposure assumption in a risk assessment will protect far more than 95% of consumers since even the most enthusiastic consumer will not consume a high level of the same product for most of their lifetime, have low body weight, absorb 100% of each substance, and be particularly sensitive to the chemical of interest.

While habits and practices (H&P) data for cannabis concentrate vaporization are limited, there are sufficient data to provide a reasonable exposure estimate (Table 3). Overall, data suggest a median/average consumption of 5–151 mg per day and a 95th percentile range of 57–140 mg/day when non-use days are considered. Importantly, all estimates for cannabis concentrate vaporization are significantly lower (more than 10-fold) than those for ENDS, which are estimated to be several grams per day [80,81]. Considering the basic tenet of toxicology and risk (i.e., “the dose makes the poison”), this lower consumption level suggests less risk from the same intentionally added substance in cannabis concentrate vaporization than ENDS use.

In 2022, the Blinc Group and LabStat published a white paper based on 1000 Canadian and 1000 American individuals that use cannabis concentrate vaporizers. Based on the average size of cartridge and duration of use, average daily consumption was estimated to be 56 mg per day in Canada and 64 mg per day in the U.S. [82]. A small subpopulation of cannabis concentrate users (*N* = 83 of 577 surveyed) from a cannabis vaporization survey of consumers from the U.S., the European Union, Australia, and New Zealand self-reported an average of 4600 ± 7000 mg cannabis concentrate use per month [83]. Considering the high degree of error, small sample size, and self-report nature of the study, the resultant data was given low weight for H&P determination.

**Figure 2:**
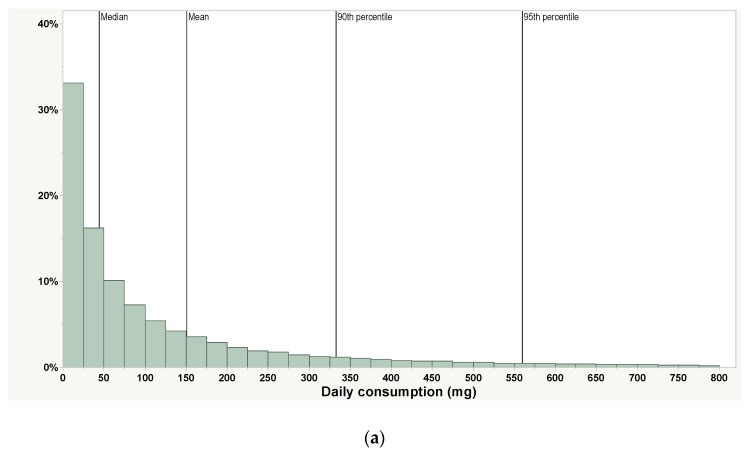

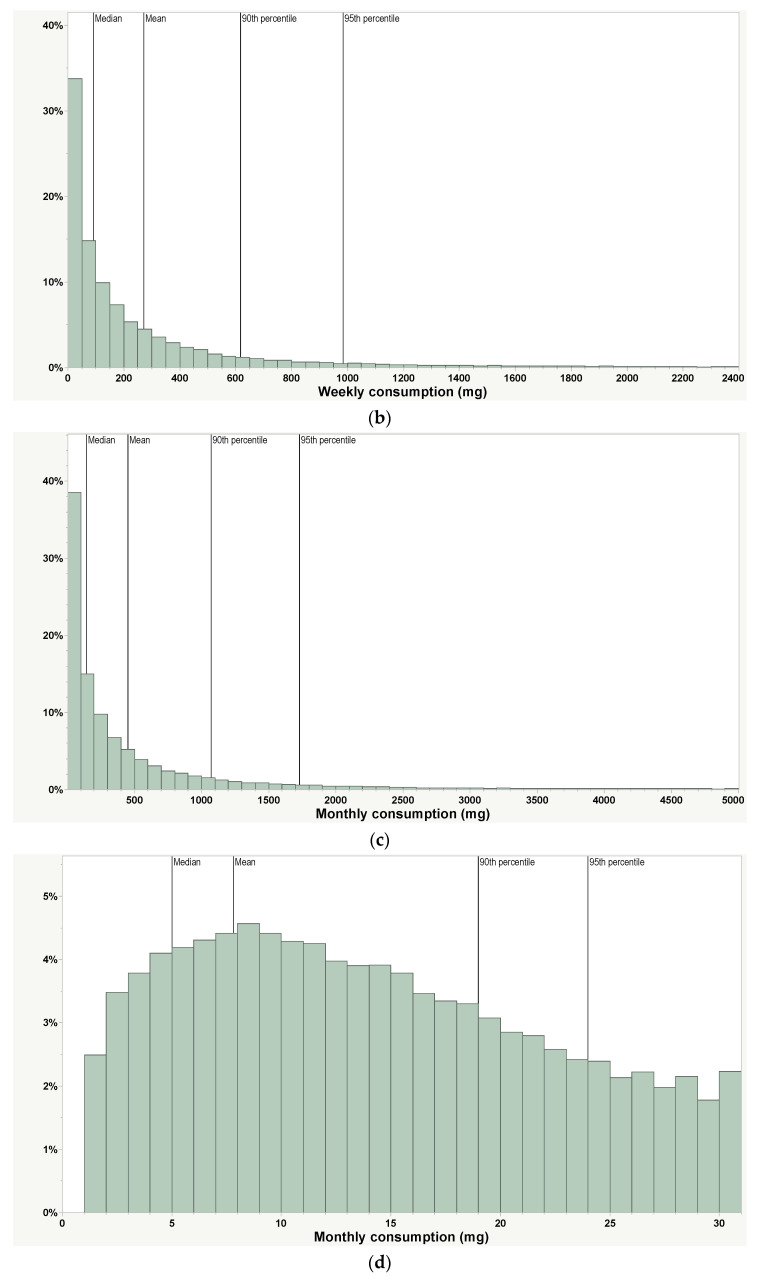
Histogram of daily (**a**), weekly (**b**), and monthly (**c**) cannabis concentrate consumption (in mg), and (**d**) use days per month from 54,500 PAX Era devices. The vertical gridlines indicate mean, median (50th percentile), 90th and 95th percentiles.

PAX^®^ Era and PAX^®^ Era Pro are closed-loop cannabis concentrate vaporizers used in regulated cannabis (THC) markets that collect anonymized usage data when users opt-in to sharing data (Table 3 and Figure 2). While these data are drawn from PAX Era devices, evidence suggests consumers titrate their THC consumption [44], so there is a strong likelihood that these consumption data apply to other cannabis concentrate vaporizers containing THC. It is unclear whether these H&P would apply to other cannabinoid vaporization products such as CBD or semi-synthetic cannabinoids (such as delta-8 or delta-10 THC). When considering data from the PAX Era App, it is important to consider daily, weekly, and monthly usage, since most consumers use their PAX devices intermittently (i.e., less than daily; Figure 2d). Surveys of convenience samples have shown that a significant portion of cannabis consumers do not consume daily [84], while others have observed that subpopulations (e.g., frequent cannabis consumers) do consume daily [85,86].

Based on THC exposure, these data align well with data published for other consumption methods. Considering the daily median or mean consumption range of 5–151 mg of cannabis concentrate (Table 3), 85% THC concentration, and 100% absorption, this suggests 4–128 mg THC/day. This aligns well with published THC consumption estimates from smoked cannabis flower [87] when considering reduced bioavailability due to side stream smoke and pyrolysis. As an example, Roternamann (2019) found the average user consumed 27.5 g of dried flower over three months, which equates to ~46 mg THC per day when 25% THC concentration and 50% delivery efficiency after pyrolysis and THC lost to side stream smoke are assumed [88,89,90,91].

The valuIs in Table 4 are recomended for exposure and risk assessment of cannabis concentrate vaporization products. These values can be used to estimate daily exposure to an additive based on a specific concentration used, adjust POD units, and determine acceptable additive concentrations in a vaporization product. Probabilistic modeling of cannabis consumption would help to refine exposure estimates further, but is not realistic for a first-tier assessment.

Daily exposure to an ingredient being evaluated is calculated by Equation (2).

(2) $$\mathrm{Daily}\;\mathrm{exposure}\;\left(\frac{\mathrm{mg}}{\mathrm{day}}\right)=\mathrm{Concentration}\;\left(\frac{\mathrm{mg}}{g}\right)\times \;\frac{\mathrm{Daily}\;\mathrm{cannabis}\;\mathrm{concentrate}\;\mathrm{consumption}\;\left(\frac{\mathrm{mg}}{\mathrm{day}}\right)}{1000}$$

where: 

Daily exposure to additive of interest: The amount of the additive in mg/day that a consumer would be exposed to at the additive concentration being evaluated.

Concentration: The concentration of the additive to be used in the cannabis concentrate formulation in mg/g. 1 mg/g = 0.1% (*w*/*w*) = 1000 ppm.

Daily cannabis concentrate consumption: Amount of the inhalable cannabis concentrate product used in 1 day for a high-level consumer. We recommend assuming 100 mg/day per Table 4.

### 3.5. Risk Characterization

Next, the exposure value calculated in Equation (2) is compared to the safety limit calculated in Equation (1). If exposure is less than the safety limit, the toxicological risk is low. If exposure exceeds the safety limit, an experienced risk assessor should conduct a more refined risk assessment to reduce unnecessary conservatism, conduct additional testing to reduce uncertainty in the risk assessment, or lower the concentration of the compound in question. Alternatively, the maximum allowable concentration of a substance in a product can be calculated with Equation (3). Equations (2) and (3) are incorporated into the Safety Limit Assessment Calculator included in the Supplementary Materials.

(3) $$\mathrm{Maximum}\;\mathrm{allowable}\;\mathrm{concentration}\;(\%\;w/w)=\frac{\mathrm{Safety}\;\mathrm{limit}\;\left(\frac{\mathrm{mg}}{\mathrm{day}}\right)}{\mathrm{Cannabis}\;\mathrm{concentrate}\;\mathrm{use}\;\left(\frac{\mathrm{mg}}{\mathrm{day}}\right)}\times 100$$

where:

Maximum allowable concentration of the additive of interest: This is the concentration of an additive in a finished product that is considered a low toxicological risk % *w*/*w*. 1% (*w*/*w*) = 10 mg/g = 100,000 ppm.

Safety limit: The exposure level for the additive calculated (in mg/day) to be an acceptable risk.

Cannabis concentrate use: Daily use (e.g., from Table 4) of the finished cannabis concentrate product in mg/day.

## 4. Discussion

The size and scope of the challenge in assessing toxicological risk of inhalable cannabis extracts is significant, and we acknowledge that this first-tier framework has some important limitations; however, we believe this guide is an important starting point for a first-tier screening and prioritization risk assessment for additives in inhaled cannabis concentrates.

### 4.1. Recommendations for Regulators

State regulators have generally reduced risk to cannabis consumers by restricting levels of pesticides, residual solvent, heavy metal, and microbial contaminants; although, there would be additional public health benefit if there were harmonized state or national-level guidelines [4]. However, when it comes to additives in manufactured inhalable cannabis concentrates, state regulators have either avoided specific guidelines or used other approaches to define restrictions targeted at protecting public health. A common approach has been to enact broad regulations based on classifications, such as natural versus unnatural, which do not inherently address consumer health, as discussed in the introduction. A second approach has been to create one-off bans, which can be effective with something like vitamin E acetate, but can be reactive without a grounding in good science and may lead to regrettable substitutions. For example, the diluents propylene glycol and medium chain triglycerides (MCT) were controversially portrayed in the media [93,94], which may have influenced manufacturers to substitute with the diluents such as vitamin E acetate and pine rosin, which we now know impart significant health risks [54,95]. Lastly, regulators have directed manufacturers to other regulatory limits that are not acceptable for cannabis products, such as the FDA inactive ingredients list for approved drug products [96]. Regulators can use the methodologies outlined in this proposed framework to prioritize additives for deriving individual limits. Using this guidance, state regulators will have a scientific process to create restrictions that can provide protection to consumers and encourage legal cannabis manufacturers and consumers to stay in the regulated market.

### 4.2. Recommendations for Manufacturers

In the cannabis industry, regulations trail behind market innovations. This means that manufacturers of cannabis products must create their own approach to ensure consumer safety and establish a responsible industry. Manufacturers can utilize this framework to conduct a first-pass assessment of ingredients used in their products and request that their additive suppliers comply with limits derived using this framework. By performing a risk assessment, they will have the data to support the materials they use, which will bolster consumer safety and confidence. Additionally, manufacturers can expand ingredient disclosures to include the names of materials that are intentionally added to the cannabis concentrate to empower the consumer to make informed decisions on which products they use. Finally, while not included in the scope of this manuscript, impurities and contaminants play an important role in toxicological risk so we encourage manufacturers to evaluate contaminants such as metals, solvents, pesticides, and microbial contaminants in their products even if it is not a regulatory requirement in their state. Jameson et al. (2022) have provided a great analysis of state level regulations for cannabis contaminants and the public health implications that manufacturers can use to determine what testing may be prudent if it is not required in their state [4].

### 4.3. Limitations and Future Directions

Limitations to this proposed framework include: (1) Cannabinoids are not in scope for this framework, but rather only additives such as terpenoids (botanical and cannabis-derived) and other intentionally added ingredients. (2) Interactions between the compounds in these complex mixtures are not considered due to a paucity of data and the complexity of the assessment. (3) Previous studies have established that the device used to vaporize cannabis extracts has a significant impact [15,17,24,53,97,98,99,100]. The device will affect the degradation products, chemicals leaching into the oil, temperature, and particle size that will affect absorption (and thus exposure). Cannabis manufacturers should consider testing aerosol produced from their extracts in the devices in which they are sold to further understand the risk from this interaction. Unfortunately, commercial laboratories with this testing capability are still lacking in most states. We encourage commercial laboratories to develop these methods to support consumers, regulators, and manufacturers. This framework and the confidence in assessments will be improved once test methods (both analytical and in vitro) and laboratories to run these tests become available for cannabis concentrate aerosols containing THC.

Finally, as the authors have done, collaboration and sharing data will ultimately improve consumer safety. At this time, the authors’ employers do not have a business relationship but are interested in advancing the safety of cannabis vape products. We hope this effort will be the start of further dialogue and begin to build a culture of collaboration between regulators, manufacturers, and academic researchers in the cannabis industry.

## 5. Conclusions

Despite the limitations based on publicly available research, we believe this framework will help to guide regulators and manufacturers in prioritizing ingredients in cannabis concentrates for additional evaluation to improve consumer safety. We also provide previously unpublished usage data from a smart vaporizer for risk assessors to use in both first-tier and more extensive risk assessments, and a user-friendly safety limit assessment calculator. There are intentionally many conservative assumptions that can reduce consumer risk and allow manufacturers and regulators to focus their efforts and limited resources on substances more likely to pose a toxicological risk. While more work is needed to ensure the safety of cannabis vaporization products, this first-tier risk assessment can guide regulators’ and manufacturers’ decision-making to protect consumer health in the present cannabis landscape, while minimizing regrettable substitutions, maintaining access to inhalable cannabis products in regulated markets, and encouraging producers to stay in the regulated market.

## Figures and Tables

**Table 1:** Default uncertainty factors.

Uncertainty Category			Value	Comment	References
1	Interspecies	Interspecies extrapolation	10	Extrapolation from animals to humans	[8,60,61]
2	Intraspecies	Intraspecies variation	1, 3 or 10	May be reduced to 3 or 1 if the study was conducted in a sensitive subpopulation (e.g., diseased individuals, children, elderly, etc.)	[8,60,61]
3	Route to route extrapolation	Oral to inhalation—toxicokinetics	2	If absorption data are available for inhalation or oral routes, they should be used instead. Combine with toxicodynamic differences if data are lacking.	[57,62]
		Oral to inhalation—toxicodynamics	3	Combine with toxicokinetic differences if data are lacking.	[62]
4	LOAEL to NOAEL or LOAEC to NOAEC	Used if adverse effects were observed at the lowest dose tested	10	Benchmark dose modeling is preferred instead of a UF, but benchmark dose modeling is out of scope for a Tier 1 safety assessment.	[8,60,61]
5	Duration	Subacute to chronic	10	Subacute = 14–90 days. Chronic ≥ 1 year.	[57]
		Subchronic to chronic	3	Subchronic = 90 days–1 year. Chronic ≥ 1 year.	[28,57,61]
6	Database	Database completeness	1, 3 or 10	Necessary if developmental or reproductive toxicity data are not available for a non-age-gated product (e.g., CBD in a convenience store). A UF of 3 can be used if the structural features of the substance are not suggestive of potential developmental toxicity as limits derived from repeat dose studies will suffice.	[63]

**Table 2:** Application of the threshold of toxicological concern (TTC) to vaporized cannabis concentrate additives.

Questions		No	Yes
1	Can the substance use the TTC approach?	If the substance fits into one of these categories, TTC cannot be used: Inorganic (e.g., metals) Radioactive constituents Nanoparticles Bioaccumulative (dioxin-like) Protein Polymer Aflatoxin-like Steroids TCDD and its analogs (i.e., polyhalogenated) Polycyclic amines Hydrazine, triazene, azide, azoxy Nitroso compounds Alpha-nitro furyl compounds	Everything else. Proceed to Q2.
2	Is the substance an organophosphate or carbamate?	Move to Q3	TTC of 18 µg/day
3	Is there a genotoxicity alert or other evidence of genotoxicity such as a positive Ames or micronucleus assay?	Move to Q4	Exposure > 10 years to lifetime: 1.5 µg/day 1–10 years: 10 µg/day 1–12 months: 20 µg/day <1 month: 120 µg/day [71]
4	Is the substance a natural cannabis substance or GRAS?	Move to Q5	Classify according to Cramer class and use the corresponding value: Cramer Class 1: 865 µg/day Cramer Class 2 or 3: 145 µg/day [75]
5	If the substance does not fit into the other categories above, TTC must be based on a wider range of substances (e.g., industrial chemicals, pesticides, etc.)	Acute aquatic toxicity MOA by OASIS in OECD QSAR Toolbox as defined by [75]: Basesurface narcotics: 22.39 µg/day Reactive: 4.286 µg/day	

**Table 3:** Cannabis concentrate vaporization consumption amount and frequency.

Timeframe	Mean	Percentile			*N*	Notes	Reference
		50th (Median)	90th	95th			
Daily, mg	64 (US) 56 (Canada)	-	-	-	2000 users		[82]
Daily, mg^1^	151	44.0	333	560	54,500 devices	Only includes use days. Most people use their products less than daily	PAX Era App
Weekly (daily average), mg ^1^	271 (39)	91.1 (13)	617 (88)	982 (140)	54,500 devices	Includes non-use days	PAX Era App
Monthly (daily average), mg ^1,2^	452 (15)	146 (5)	1081 (35)	1743 (57)	54,500 devices	Includes non-use days	PAX Era App
Days of consumption per month, days	7.8	5	19	24	54,500 devices		PAX Era App
Daily, mg	80	-	-	-	-	Estimated based on THC consumed per joint year	[47]
Monthly (daily average), mg ^2^	4600 +/− 7000 (150.8)	1000 (32.8)	-	-	83 users	Self-reported use from a U.S., EU, Australia, and New Zealand	[83]

^1^ 90th and 95th percentiles are likely to be significant overestimates due to the error associated with exposure estimation. See Section 2 for details. ^2^ Assumes 30.5 days per month.

**Table 4:** Exposure assumptions for risk assessment of cannabis extract vaporization additives.

Exposure Assumption	Value	Notes and References
Daily cannabis concentrate product consumption ^1^	100 mg/day	Average of 95th percentile between weekly and monthly from PAX Era device (Table 3). This is expected to be a conservative estimate of average daily use of a cannabis concentrate product for a high-level consumer. This value can be used to estimate additive exposure as indicated in Equation (2)
Bioavailability	100%	Conservative assumption
Body weight	60 kg	Conservative body weight assumption for American adult female. This would be more conservative than assuming an adult male of higher body weight
Inhalation volume: 1 full day (24 h)	20 m^3^	Average daily inhalation rate [27,28,29]
Inhalation volume: 8 h or 1 workday	6.7 m^3^	Average inhalation rate over a workday [27,28,29]
Inhalation volume: 15 min	0.3 m^3^	Average inhalation over 1 day or 1 h adjusted for time [28,92]

^1^ For THC-containing inhalable cannabis concentrate products. CBD, synthetic, and semi-synthetic cannabinoid products are not included.

## Data Availability

The underlying datasets presented in this study are openly available in FigShare at doi: 10.6084/m9.figshare.21699470.

## Supplementary Materials

The following supporting information can be downloaded at: https://www.mdpi.com/article/10.3390/toxics10120771/s1: Case Study S1: Case Study Using Toxicological Data on the Compound of Interest; Case Study S2: A Case Study Using Occupational Exposure Levels (OELs) to Assess Risk; Case Study S3: A Case Study Using TTC. Safety Limit Assessment Calculator (SLAC) which computes the safety limit based on chosen PODs and UFs.

## Selected Abbreviations and Definitions

ACGIH	American Conference of Governmental Industrial Hygienists
Adverse effect	A harmful effect. Examples include a change in morphology or impairment of function.
CBD	Cannabidiol, a non-intoxicating phytocannabinoid in cannabis
Concentration	The level of a substance of interest in a medium (e.g., air, cell culture media, or solvent). Units may be in %, mg/L, or mg/m^3^ of air.
Dose	The exposure to a cannabis concentrate consumer (in mg per day or mg/kg body weight per day) or the test species in a toxicological study.
ECHA	European Chemicals Agency
EFSA	European Food Safety Authority
ENDS	Electronic nicotine delivery systems
EPA	U.S. Environmental Protection Agency
FEMA	Flavor and Extract Manufacturers Association of the U.S. FEMA is a trade organization supporting the U.S. FDA by establishing the GRAS status of food substances.
GCP	Good clinical practice. Guidelines are laid out in ICH E6 [101] to assure the quality and integrity of clinical studies.
GLP	Good laboratory practice—a set of principles to assure quality and integrity of non-clinical studies laid out in 21 CFR 58.
GRAS	Generally recognized as safe in food under certain conditions of use. GRAS status alone cannot demonstrate safety for inhalable cannabis concentrate additives; however, the underlying data to determine GRAS status may be useful in the risk assessment.
IFRA	International Fragrance Association
LOAEL	Lowest adverse effect level. The dose at which an adverse biological response is first observed.
NAMs	New approach methodologies. These are approaches used in risk assessment to fill information gaps that aim to reduce animal use. This generally includes in vitro, in chemico, and in silico.
NIOSH	U.S. National Institute for Occupational Safety & Health
NOAEC	No adverse effect concentration. The concentration at which no adverse effects are observed, typically in an inhalation study in units of mg/m^3^ or ppm.
NOAEL	No adverse effect level. The dose at which no adverse effects are observed. This is typically in units of mg/kg.
OEHHA	California Office of Environmental Health Hazard Assessment
OSHA	U.S. Occupational Safety and Health Administration
POD	Point of Departure. The dose chosen as a basis for making extrapolations needed for assessing risk, usually the NOAEL or LOAEL.
RIFM	Research Institute for Fragrance Materials
TCEQ	Texas Commission on Environmental Quality
THC	Delta-9 tetrahydrocannabinol, the substance in cannabis generally responsible for the intoxicating effect.
Toxicodynamics	The biological effects of a substance
Toxicokinetics	The disposition of substances in the body, including absorption, distribution, metabolism, and elimination.
TTC	Threshold of toxicological concern
WHO	World Health Organization
