# Supplementary material for: A First-Tier Framework for Assessing Toxicological Risk from Vaporized Cannabis Concentrates
Source: Toxics. 2022 Dec 9;10(12):771. doi: 10.3390/toxics10120771 (PMC9782653; doi:10.3390/toxics10120771)
Supplement: Supplementary file 1 [file toxics-10-00771-s001.zip › toxics-2011150-supplementary.pdf]

toxics MDPI

Supplementary Materials: A First-Tier Framework for Assessing Toxicological Risk from Vaporized Cannabis Concentrates

Shawna Vreeke, David M. Faulkner, Robert M. Strongin and Echoleah Rufer

Case Study S1: Case Study Using Toxicological Data on the Compound of Interest

Diesel-2-gelatomine is not naturally present in cannabis, is not mutagenic based on negative guideline-compliant Ames and micronucleus studies and is not known to be a respiratory sensitizer or developmental toxicity. It has an OECD 408 subchronic study (GLP) in which rats were gavaged with 0, 10, 30, and 100 mg/kg diesel-2-gelatomine (95% purity) 5 days per week for 90 days. Liver toxicity was observed at 100 mg/kg diesel-2-gelatomine and no adverse effects were observed at 30 mg/kg diesel-2-gelatomine. No additional toxicity data are available. Thus, the NOAEL of 30 mg/kg was used as the POD.

$$\text{Safety limit} = \frac{POD}{UF1\ x\ UF2\ x\ UF3\ x\ UF4\ x\ UF\ 5\ x\ UF6} = (1)$$

$$\frac{30\frac{mg}{kg}x\ 95\%\ purity\ x\ \frac{5}{7}days\ per\ week}{10\ x\ 10\ x\ (2x3)x\ 1\ x\ 3x\ 1} = (2)$$

0.1 mg diesel-2-gelatomine per kg of body weight or 0.6 mg per day

where:

UF1: Interspecies extrapolation from rat to human is 10.

UF2: Intraspecies extrapolation for sensitive individuals is 10.

UF3: 6 total to account for 2 for oral to inhalation extrapolation for toxicokinetic considerations since no absorption data are and 3 for toxicodynamic considerations since no inhalation data are available.

UF4: LOAEL to NOAEL is 1 since a NOAEL was found in the critical study used to define the POD.

UF5: Extrapolation from subchronic rodent study to potential lifetime exposure for consumers is 3.

UF6: 1 since this is not a developmental toxicant.

Considering 60 kg body weight, exposure less than 0.6 mg per day for an adult is considered a negligible risk. This was calculated by multiplying the safety limit of 0.1 mg/kg x 60 kg. Based on this safety limit, a maximum concentration of 0.6% w/w in a cannabis concentrate or less is considered low risk.

$$\text{Diesel-2-gelatomine allowable concentration (\% w/w)} = \frac{safety\ limit\ (0.6\ mg)}{daily\ exposure\ (100\ mg)} \times 100 = 0.6\% \quad (3)$$

A company would like to use diesel-2-gelatomine at 0.5% in a cannabis concentrate with high levels of THC. Since this is below the maximum allowable concentration, it is considered a low risk for use in that product. In vitro studies to understand local respiratory toxicity and analytical studies to measure possible degradation products would help to further evaluate and potentially reduce the risk associated with vaporization of this compound.

Case Study S2: Case Study Using Occupational Exposure Levels (OELs) to Assess Risk

A manufacturer of cannabis concentrate vape cartridges with a high level of THC adds a blend of terpenes that contains para-cymene to the extract for a final concentration of 1.05% w/w. The Nordic countries have derived a time-weighted average threshold limit value (TWA-TLV) of 25 ppm (135 mg/m^3) for para-cymene [1]. Para-cymene is not expected

to be a developmental toxicant. The manufacturer can use the occupational exposure limit to analyze the risk posed by para-cymene.

$$POD = TWA - TLV\ x\ volume\ of\ inhalation\ over\ 8\ hours\ x \frac{5\ days}{7\ days} \quad (4)$$

$$= \frac{135mg}{m^3}\ x\ 6.7\ m^3\ x \frac{5}{7} = 646\ mg/day \quad (5)$$

The TWA-TLV would be equivalent to an exposure of 646 mg of p-cymene in a day.

$$\text{Safety limit} = \frac{POD\ (646\ mg)}{UF\ (1\ x\ 10\ x\ 1\ x\ 1\ x\ 3\ x\ 1)} = 21.5\ \text{mg} \quad (6)$$

where:

UF1: 1 since the TWA-TLV is meant for humans, interspecies extrapolation is not needed.

UF2: 10 to account for extrapolation for sensitive individuals.

UF3: 1 since the regulation is for inhalation exposure. No extrapolation is needed.

UF4: 1 since the TWA-TLV would be comparable to a NOAEL – the level at which no adverse effects would be expected.

UF5: Extrapolation to potential lifetime exposure for consumers is 3.

UF6: 1 since this is not a developmental toxicant.

Based on Equation 3, the allowable concentration can be calculated by:

$$\text{Allowable concentration (\% w/w)} = \frac{safety\ limit\ (21.5\ mg)}{daily\ exposure\ (100\ mg)} \times 100 = 21.5\% \quad (7)$$

Using the assumption that 100 mg of the product is inhaled, the allowable concentration for para-cymene has resulted in 21.5% (w/w). Since the current use limit of 1.05% is less than the proposed allowable limit of 21.5%, para-cymene may be assumed to impart negligible risk at this concentration.

Allowable concentration > current use concentration so it is Acceptable for use as proposed

Although this concentration may well pose little toxicological risk, a high level of a flavor compound is unlikely to be desirable from a sensorial standpoint. In vitro studies to understand local respiratory toxicity and analytical studies to measure possible degradation products would help to further reduce the potential risk from this compound.

Case Study S3: Case Study Using TTC

Highlyronic acid is a compound not normally found in cannabis that is not excluded from TTC per Table 2, Q1. It is not an organophosphate or carbamate (Table 2, Q2). There is a negative Ames test for highlyronic acid and no structural alerts for genotoxicity (Table 2, Q3 and Figure S1), so the Cramer decision tree can classify the substance. However, since highlyronic acid is neither a natural cannabis substance nor GRAS, the Cramer decision tree may not be sufficiently conservative to protect consumer health, given the lack of testing data (Table 2, Q4). Therefore, the Nelms & Patlewicz TTC values are more appropriate since they are calculated based on a wider range of substances and are more conservative. (Nelms & Patlewicz, 2020)

The Nelms & Patlewicz TTC values are determined by the observed acute aquatic toxicity mechanism of action, and this may be derived using the Verhaar Scheme decision tree, which may be found in the OECD QSAR Toolbox or the Toxtree web application (Toxtree), or OASIS, which is also in the OECD QSAR Toolbox. Entering the structure or CAS number for highlyronic acid into the Verhaar scheme tool indicates that this compound is in the first TTC category for Nelms & Petlewicz (Fig S2), and therefore, we can use the TTC value of 22.39 µg/day (0.022 mg/day).

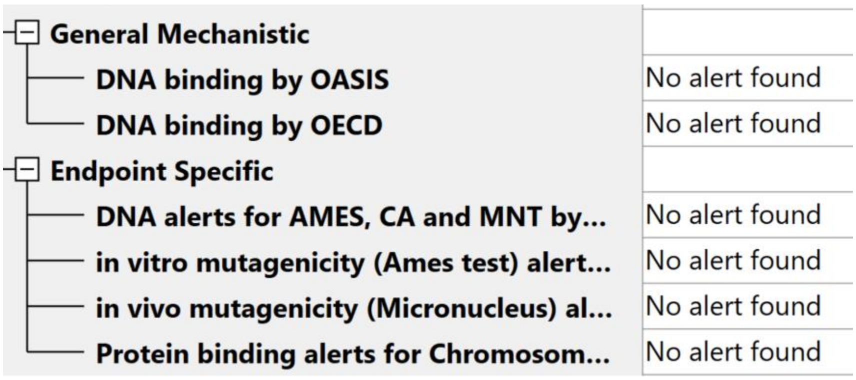

Figure S1. Screenshot from OECD QSAR Toolbox showing that highlyronic acid does not have any structural alerts for genotoxicity (indicated by DNA binding).

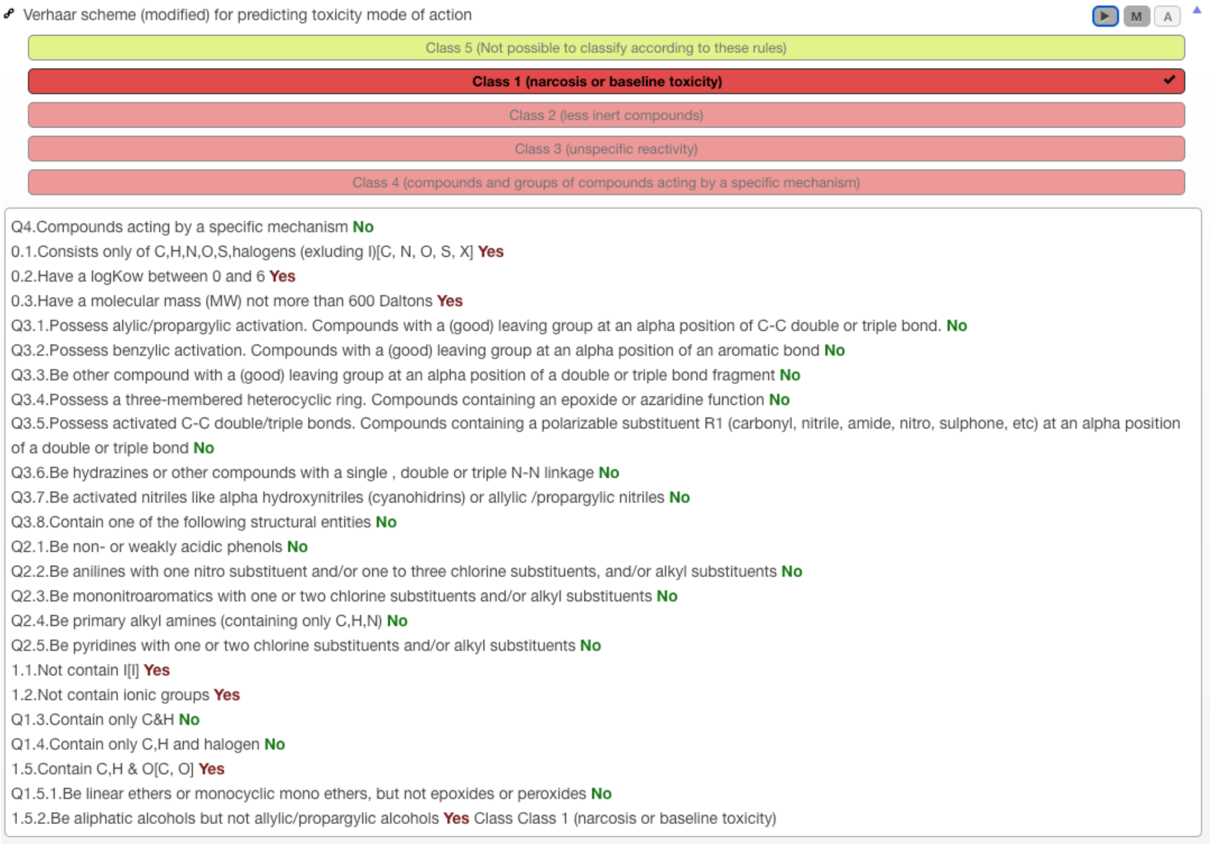

Figure S2. Highlyronic acid classification as shown in Tox Tree. The class is indicated based on the classification with a checkmark at the top of the output.

Based on Equation 3, the allowable concentration can be calculated by:

$$\text{Allowable concentration (\% w/w)} = \frac{safety\ limit\ (0.022\ mg)}{daily\ exposure\ (100\ mg)} \times 100\ = 0.022\%$$

A manufacturer would like to use highlyronic acid at 0.3% in a cannabis concentrate containing a high concentration of THC.

Allowable concentration < proposed use concentration

There are three options the manufacturer can take:

Remove highlyronic acid or reduce concentration in the product

Engage a toxicologist to determine whether a higher tier assessment may allow higher concentration.

Conduct testing that will support a higher level of use.

References

1. NIOSH. *p-cymene*. 2017. https://www.cdc.gov/niosh-rtecs/gz5aca30.html (accessed 5 August 2022).
2. *E6(R2) Good Clinical Practice*; ICH: Geneva, Switzerland, 2016. https://www.ich.org/page/efficacy-guidelines, accessed on 27 October 2022.
